# Evaluation of LipL32 and LigA/LigB Knockdown Mutants in *Leptospira interrogans* Serovar Copenhageni: Impacts to Proteome and Virulence

**DOI:** 10.3389/fmicb.2021.799012

**Published:** 2022-02-02

**Authors:** Luis G. V. Fernandes, Ellie J. Putz, Judith Stasko, John D. Lippolis, Ana L. T. O. Nascimento, Jarlath E. Nally

**Affiliations:** ^1^Infectious Bacterial Diseases Research Unit, USDA Agricultural Research Service, National Animal Disease Center, Ames, IA, United States; ^2^Laboratório de Desenvolvimento de Vacinas, Instituto Butantan, São Paulo, Brazil; ^3^Ruminant Diseases and Immunology Research Unit, USDA Agricultural Research Service, National Animal Disease Center, Ames, IA, United States

**Keywords:** *Leptospira*, leptospirosis, CRISPR interference, LipL32, LigB, virulence, hamster, LigA

## Abstract

Leptospirosis is a worldwide zoonosis caused by pathogenic species of the genus *Leptospira*. The recent application of CRISPR interference (CRISPRi) to *Leptospira* facilitates targeted gene silencing and provides a new tool to investigate pathogenic mechanisms of leptospirosis. CRISPRi relies on the expression of a catalytically “dead” Cas9 (dCas9) and a single-guide RNA (sgRNA). Previously, our group generated a LipL32 and a double LigA/LigB (LigAB) mutant, which, in the current study, are characterized by whole-cell proteomics in comparison with control leptospires harboring plasmid expressing dCas9 alone. Comparison of control and LigAB mutant leptospires identified 46 significantly differentially expressed (DE) proteins, including 27 proteins that were less abundant and 19 proteins that were more abundant in the LigAB mutant compared with the control. Comparison of the control and LipL32 mutant leptospires identified 243 DE proteins, of which 84 proteins were more abundant and 159 were less abundant in the LipL32 mutant strain. Significantly increased amounts of known virulence impactors and surface membrane receptors, including LipL45, LipL31, LigB, and LipL41, were identified. The virulence of LipL32 and LigAB mutants were evaluated in the hamster model of leptospirosis; the LigAB mutant was unable to cause acute disease although mutant leptospires could still be recovered from target organs, albeit at a significantly lower bacterial burden (<850 and <16-fold in liver and kidney, respectively, in comparison with control), indicating attenuation of virulence and a shift to chronic bacterial persistence. Notably, the LipL32 mutant displayed augmented virulence as evidenced by early onset of clinical symptoms and increased numbers of circulating foamy macrophages. Validation of LipL32 and LigAB mutants recovered from liver and kidney in the presence or absence of antibiotic selection revealed high plasmid stability and, by extension, gene silencing *in vivo*. Collectively, this work emphasizes the advantages and feasibility of using CRISPRi technology to evaluate and characterize virulence factors of leptospires and their respective host–pathogen interactions in animal models of leptospirosis. Importantly, it also provides insight into the requirements of LigA and LigB for acute disease and explores the impact of silencing expression of *lipL32*, which resulted in substantial changes in amounts of outer membrane proteins.

## Introduction

Leptospirosis is a neglected zoonosis caused by pathogenic and virulent species of the genus *Leptospira*, responsible for more than one million cases and almost 60,000 human deaths per year worldwide (Bharti et al., 2003; Adler, 2010; Costa et al., 2015). Rodents are widespread asymptomatic reservoirs and the main carriers of the disease in urban areas, harboring leptospires in the proximal tubules of their kidneys (Putz and Nally, 2020). Humans and other animals can be infected through skin abrasions or exposed mucosa *via* contact with urine of infected hosts either directly or indirectly through contaminated water or soil (Adler, 2010), and infection can result in a wide variety of symptoms, ranging from non-specific symptoms, such as fever, chills, headache, and myalgia, to severe leptospirosis, a potentially fatal condition known as Weil’s disease (Levett, 2001; Bharti et al., 2003). In addition, severe pulmonary hemorrhage leptospirosis (SPHL) (Dolhnikoff et al., 2007) or severe pulmonary hemorrhagic syndrome (Ko et al., 1999; Segura et al., 2005; Maciel et al., 2008) is recognized as an emerging clinical manifestation over the last two decades and the main cause of death in some epidemics (Bharti et al., 2003; Nally et al., 2004).

Before reaching target organs, leptospires need to overcome host immune barriers and reach the bloodstream, where they must escape the bactericidal effect of the complement system. It has been previously shown that pathogenic *Leptospira* can express surface exposed adhesins responsible for binding to extracellular matrix (ECM) (Vieira et al., 2014; Fernandes et al., 2016) and components of plasma, including plasminogen, fibrinogen, thrombin, complement molecules, and immune regulators that can subvert the host response to infection (Verma et al., 2006; Barbosa et al., 2009; Oliveira et al., 2013; Fernandes et al., 2015; Kochi et al., 2019). Leptospiral immunoglobulin-like proteins LigA and LigB have been extensively studied *in vitro* and interact with a broad range of host ligands, including fibronectin, laminin (Choy et al., 2007), and elastin (Lin et al., 2009), thus highlighting their significance in the initial stages of infection. Further, Lig proteins interact with the negative complement regulators Factor H and C4bp (C4-binding protein) (Castiblanco-Valencia et al., 2012), suggesting that they facilitate resistance to serum and as demonstrated with pathogenic species *in vitro* (Cinco and Banfi, 1983; Meri et al., 2005; Fraga et al., 2016). In addition, heterologous expression of Lig proteins in the surrogate saprophytic *L. biflexa* cells resulted in the increased ability to survive complement killing *in vitro* (Castiblanco-Valencia et al., 2016). When evaluated in the hamster model of infection, mutants of LigB alone (Croda et al., 2008) displayed no effect upon leptospiral virulence, likely due to the redundancy displayed by multiple Lig proteins (Haake and Matsunaga, 2020). However, incomplete knockdown of both LigA and LigB by transposon-delivered transcription activator-like effectors targeting the promoter region of both genes impacted the virulence of *L. interrogans* serovar Manilae in the hamster model (Pappas and Picardeau, 2015) because two out of three mutants were avirulent and not recovered from animal tissue.

LipL32 is an outer membrane lipoprotein found exclusively in pathogenic *Leptospira* spp. It is the most abundant surface membrane protein (Haake et al., 2000; Malmström et al., 2009) and a commonly used marker of detection and diagnostics (Guerreiro et al., 2001). Despite the extensive range of host ligands that can interact with LipL32, including several ECM proteins, such as laminin, collagen, fibronectin, and the plasma proteins plasminogen and fibronectin [reviewed by Murray (2013)], a transposon mutant knocked out for *lipL32* displayed no attenuation of virulence in animal models nor affected interaction with host molecules *in vitro* (Murray et al., 2009). The role of the major outer membrane LipL32 protein remains enigmatic, yet understanding this role is important for one of the most abundant outer membrane proteins in the repertoire of pathogenic *Leptospira*.

The demonstration of CRISPR interference (CRISPRi) for leptospiral mutant generation, in addition to the use of Hornsby-Alt-Nally (HAN) media (Fernandes et al., 2021) for rapid colony recovery, has enabled a new generation of tools for application to understand pathogenic mechanisms of leptospirosis. The creation of knockdown mutants could allow the confirmation of native protein function, previously only extrapolated by assays with recombinant proteins as per the demonstration of the augmented susceptibility to complement killing displayed by a LigA/LigB double knockdown mutant (Fernandes et al., 2021). CRISPRi applied to *Leptospira* spp. in its current state relies on the episomal expression of two components: a DNA binding protein, which is catalytically inactive Cas9 (dCas9), and a single-guide RNA, which targets a gene for silencing by base pairing. Although high plasmid stability was shown *in vitro*, the evaluation of knockdown mutants during animal experiments remains to be demonstrated.

In this work, knockdown LipL32 and double LigA/LigB (LigAB) mutants are proteomically characterized and evaluated in the hamster model of infection. Collectively, these results demonstrate the applicability of CRISPRi-generated mutants for evaluation of virulence *in vivo*.

## Materials and Methods

### Mutant *L. interrogans* Strains

Virulent, low passage *L. interrogans* serovar Copenhageni strain Fiocruz L1-130 were transformed by conjugation with conjugative *E. coli* β2163 cells containing the plasmids pMaOri.dCas9sgLipL32 or pMaOri.dCas9sgLigAB for silencing of LipL32 and both LigA and LigB, respectively. As a no-silencing control, pMaOri.dCas9 was also used. Protospacers contained in sgRNA cassettes to target genes encoding LipL32, LigA, and LigB, conjugation protocols and mutant recovery were previously described by Fernandes et al. (2021). Mutants were confirmed by PCR with primers pMaOri2 F (5′ACGCAATGTATCGATACCGAC 3′) and R (5′ATAGGTGAAGTAGGCCCACCC 3′) flanking the sgRNA cassette and by immunoblotting of cell extracts (5 × 10^7^ cells/lane) with rabbit polyclonal anti-LipL32, anti-LigAB Fernandes and anti-LipL41 (loading control). Lipopolysaccharide was visualized by staining bacterial cell extracts (5 × 10^7^ cells) with Pro-Q Emerald 300 (Invitrogen, CA, United States) as per the manufacturer’s guidelines. For downstream experiments, including animal infection, all mutants, and wild type containing pMaOri.dCas9 without sgRNA, were synchronized regarding their *in vitro* passage and conjugation experiments.

### Whole-Cell Proteomics of *L. interrogans* Mutants

Cultures of *L. interrogans* strain Fiocruz L1-130 LipL32 and LigAB mutants, along with leptospires containing the empty pMaOri.dCas9 plasmid, were grown until they reached mid-late log growth phase (∼2 × 10^8^ cells/mL). Concurrent with the preparation of bacterial cells for hamster challenge, 10 mL of each bacterial culture was counted and centrifuged (10,000 × *g*, 4°C, 30 min), washed twice with ice-cold TE buffer, and the resulting pellet frozen at −80°C until analysis. Pellets were solubilized overnight in 7 M Urea, 2 M Thiourea, and 1% ASB-14 and then reduced and alkylated with DTT and IAA, respectively. Samples were then diluted with 50 mM Tris–Cl pH 8.0 to reduce final concentration of urea to less than 1 M. Protein concentrations were determined using the RC DC protein assay (BioRad, CA, United States). Samples were digested overnight with Trypsin/Lys-C Mix as per the manufacturer’s instructions (Promega Corporation, WI, United States). Digested samples were run through a desalting Pierce C18 spin column (Thermo Fisher Scientific, FL, United States) per the manufacturer’s specifications. Dried samples (minimum 25 μg, equal across all samples) were resuspended in a 95% H_2_O 5% acetonitrile and 0.1% formic acid solution and submitted to the LTQ OrbiTrap Velos Pro (Thermo Fisher Scientific, FL, United States) mass spectrophotometer. The Thermo Dionex UltiMate 3000 RSLCnano system (Thermo Fisher Scientific, FL, United States) was used for peptide chromatography. An Acclaim PepMap 100 C18 column was used to separate peptides with a mobile gradient after which they were fed into a Nanospray Ion Source (Thermo Fisher Scientific, FL, United States). Raw data was processed using MaxQuant software (version 1.6.7.0) (Cox and Mann, 2008), using a FASTA database downloaded from UniProt with the search terms of leptospira + interrogans + strain Fiocruz (downloaded Sept. 21, 2020). Data from MaxQuant is then further processed using Perseus (version 1.6.14) (Tyanova et al., 2016). Hawaii plots were generated using Perseus and used to determine differentially expressed proteins. Proteins were considered significantly differentially expressed with an FDR < 0.05.

A search for possible off-targets to each sgRNA within *L. interrogans* serovar Copenhageni strain Fiocruz L1-130 that could impact proteomes was performed by the server Cas-OFFinder (Bae et al., 2014) with up to five mismatches allowed.

### Animal Ethics Statement

All animal experimentation was conducted in accordance with protocols as reviewed and approved by the Animal Care and Use Committee at the National Animal Disease Center, and as approved by USDA Institutional guidelines. Weaned female hamsters were acclimated to the facility a week prior to challenge at 4–5 weeks of age. Hamsters were monitored daily and always had *ad libitum* access to food and water.

### Leptospirosis Challenge

The results presented herein are from two separate animal experiments. In the first experiment, LipL32 and LigAB mutants (third passage) were cultured to mid-late log phase in HAN media at 29°C. Groups of recently weaned, 4− to 5-week old female Syrian hamsters (*Mesocricetus auratus*, group *n* = 4, weighing 52.56 ± 8.56 g) were inoculated intraperitoneally (IP) with 10^8^ of distinct and passage-synchronized mutants of leptospires or with 1 mL HAN media (negative control). Each animal was monitored daily and weighed for clinical signs of acute leptospirosis. One consistently identifiable feature of *L. interrogans* serovar Copenhageni strain Fiocruz L1-130 infection is weight loss displayed by moribund animals; therefore, animals were humanely euthanized when weight loss (>10%) and/or additional clinical signs (blood on paws/nose/urogenital tract, lethargy, etc.) were observed or based on experimental time points. Once the augmented and attenuated virulence phenotypes were observed for the LipL32 and LigAB mutant challenges, respectively, a second animal experiment was conducted with additional and alternative experimental endpoints. For the second experiment, 4-week-old animals (weighting 57.31 ± 5.28 g) were inoculated with 10^8^ leptospires, comprising third passage mutant leptospires cultured from kidney macerates in the first animal experiment. Endpoints of animals inoculated with LipL32 or LigAB silenced strains were compared with those displayed by animals infected with *L. interrogans* containing empty pMaOri.dCas9 plasmids by the log-rank test, and a *p*-value below 0.05 was considered statistically significant.

### Endpoint Culture and Hamster Sample Collection

In this study, acute disease is defined by the appearance of severe clinical symptoms in the first 7 days following experimental challenge, whereas chronic disease is defined as the absence of severe clinical symptoms coupled with establishment of bacterial colonization after a minimum of 2 weeks post disease challenge. Severe clinical symptoms of leptospirosis in hamsters include weight loss (> 10%), blood on paws/nose/urogenital tract, lethargy, poor hygiene, and others. Once severe clinical symptoms appeared, or when hamsters reached an experimental time point (see Table 1), animals were humanely euthanized as previously published (Zuerner et al., 2012). One kidney and one liver lobe were harvested and immediately macerated in 9 mL of HAN media plus 5-fluorouracil (5-FU). Suspensions were used at different dilutions to inoculate HAN media plus 5-FU without or with spectinomycin (to select for the pMaOri.dCas9 backbone) to confirm mutant identities and intact plasmids. Cultures were monitored daily by dark-field microscopy. An additional section of kidney and lobe of liver were harvested and frozen at −80°C for bacterial burden analysis.

**TABLE 1:** Detailed description of animal necropsy and sample collection.

Animal experiment one		
Group	Number of animals during acute time frame (≤ 7 days post challenge)	Number of animals during chronic time frame (days 14–22 post challenge)

Media Alone Negative Controls	Two hamsters (day 5, Asymptomatic)	Two hamsters (day 19, Asymptomatic)
pMaOri.dCas9 Only	Four hamsters (day 5, Clinical Symptoms)	NA
LipL32 mutant	Four hamsters (two at day 4, two at day 5, Clinical Symptoms)	NA
LigAB mutant	NA	Four hamsters (day 19, Asymptomatic)
**Animal Experiment Two**		
Group	Number of animals during acute time frame (≤ 7 days post challenge)	Number of animals during chronic time frame (days 14–22 post challenge)
Media Alone Negative Controls	Six hamsters (three at day 5, three at day 6, Asymptomatic)	Two hamsters (day 19, Asymptomatic)
pMaOri.dCas9 Only	Eight hamsters (four at day 5, four at day 6, Clinical Symptoms)	NA
LipL32 mutant	Four hamsters (day 5, Clinical Symptoms)	NA
LigAB mutant	Four hamsters (day 6, Asymptomatic)	Four hamsters (day19, Asymptomatic)

*Clinical Symptoms’ denote animals that presented with known severe signs of disease including weight loss, blood on nose/urogenital tract, lethargy, poor condition, etc.*

*“Asymptomatic” labels denote animals that lacked any signs of disease, but samples were collected based on experimental design.*

*NA, Not-applicable’ as no animals were collected at this timepoint.*

At the time of euthanasia, hamsters additionally had whole blood collected with needle and syringe by cardiac puncture as previously reported (Putz et al., 2021). Blood smears were instantaneously made and dried overnight. Whole blood slides were subsequently stained with May-Grunwald Giemsa as described (Putz et al., 2021). Coverslipped slides were then manually evaluated to determine morphology and count based on a 100-cell differential leukocyte analysis. Cell counts were evaluated in R (version 3.6.1) using simple regression models and treating the challenge strain as a fixed effect. Pairwise comparisons were used to evaluate differences between challenge strains and *p*-values ≤ 0.05 were considered significant. Least squares means and standard errors are reported. Acute and chronic time point data were combined for the LigAB mutant observations.

### Quantification of Bacterial Load on Target Organs

Approximately 15–20 mg of kidney cortex and liver tissue were used for DNA isolation using the DNeasy DNA Blood and Tissue isolation kit (Qiagen, MD, United States) following the manufacture’s specifications and using a final elution volume of 200 μL. For composing a concentration curve of leptospiral genomic equivalent (GEq), genomic DNA from *L. interrogans* was extracted using the same kit; genome size and composition were used to infer the absolute mass per genome. The bacterial load on target organs was quantified by a TaqMan-based quantitative-PCR assay using an CFX384 Real-Time System (Biorad, CA, United States). The pathogen-specific *lipL*32 gene was amplified in the samples (5 μL template/well) using primers described previously (Wunder et al., 2016), LipL32F (5′AAGCATTACCGCTTGTGG TG 3′) and LipL32R (5′ GAACTCCCATTTCAGCGATT 3′), and amplicon of 242 bp was detected by the probe, LipL32-189P [6-carboxyfluorescein (FAM)-5′AAAGCCAGGACAAGCGCCG 3′-black hole quencher 1 (BHQ1)]. Total volume of PCR reactions was 25 μL and consisted of 400 nM of each primer and 132.5 nM of the specific probe. The amplification protocol consisted of 2 min at 50°C and 10 min at 95°C, followed by 45 cycles of amplification (95°C for 15 s and 60°C for 1 min). A result was considered negative if no amplification occurred or if the Ct was greater than 40. The concentration of leptospires, expressed as GEq per gram of tissue, was calculated based on the standard curve equation.

Statistical differences between groups of interest were determined by one-tailed Mann–Whitney non-parametric test and *p*-values ≤ 0.05 were considered statistically significant.

### Target Protein Expression in Recovered Mutants

Leptospires cultured out of HAN media after organ macerates inoculation with or without spectinomycin (40 μg/mL) were harvested from the media by centrifugation (10,000 × *g*, 30 min, 4°C), washed twice with PBS, and then suspensions were counted and normalized to 5 × 10^9^ cells/mL. Protein samples were then processed for one-dimensional (1-D) SDS-PAGE on 12% acrylamide gels (BioRad) as per manufacturer’s guidelines. Immunoblots were performed to infer the maintenance of gene silencing as previously described (Fernandes et al., 2021).

## Results

### Validation of Mutants

**FIGURE 1:**
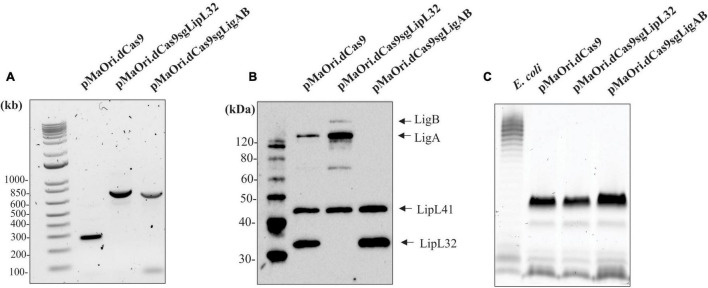
Confirmation of knockdown LigAB and LipL32 mutagenesis. **(A)** Depicts the presence of the empty pMaOri.dCas9 plasmid and the cassettes containing the sgRNA targeting *lipL32* and *ligAB* by PCR with primers flanking the sgRNA cassette. **(B)** Representative immunoblot of 5 × 10^7^ recombinant cells containing empty pMaOri.dCas9 plasmid alone or with sgRNA for LipL32, and LigAB, which have been probed with antisera specific for LigA/B, LipL41, and LipL32. **(C)** Staining for LPS in 5 × 10^7^ recombinant cells containing empty pMaOri.dCas9 plasmid alone or with sgRNA for LipL32, and LigAB. LPS from *E. coli* serotype 055:B5 was used as a positive control. Molecular mass markers (M) (kDa) are indicated in panels **(A,B)**.

Prior to animal infection, mutants obtained from the same conjugation experiment and at the same passage number were evaluated for the presence of the plasmids coding the CRISPRi system and for the silenced expression of the target proteins. Amplicons obtained after PCR with primers flanking the sgRNA cassette (Figure 1A) and immunoblotting of the whole-cell extracts (Figure 1B) confirm that all the strains were recombinant and target proteins were absent. Interestingly, LigA and LigB expression appears to be increased in the LipL32 mutant, and this observation was consistent through all mutant immunoblots (Supplementary Figure 1). The classic component of Gram-negative bacteria and toll-like receptor (TLR) agonist, lipopolysaccharide (LPS), has a similar profile and concentration among all strains (Figure 1C).

### Proteomic Analysis

To further characterize LigAB and LipL32 mutant strains, we utilized peptide chromatography and mass spectrometry analysis. Proteomic samples were prepared from LigAB and LipL32 mutant leptospire cultures as well as from leptospires containing the empty pMaOri.dCas9 plasmid alone as a control. In total, 1,215 proteins were identified in our samples using the *L. interrogans* strain Fiocruz L1-130 reference. Of those, 948 proteins were identified by more than one unique peptide, and expression differences between mutant and empty plasmid control preparations were determined. In total, from those 948 proteins, 46 proteins were differentially expressed for LigAB mutant samples with a false discovery rate (FDR) of 0.05, and 243 proteins for LipL32 mutants (Supplementary Table 1).

**FIGURE 2:**
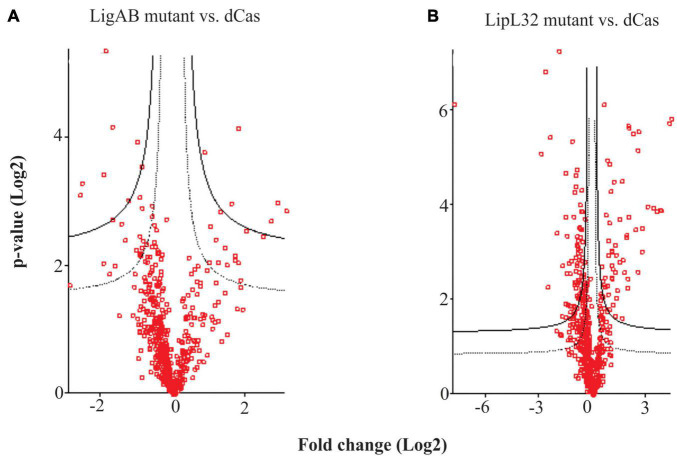
Hawaii plots showing differential expression of proteins between mutant and empty dCas9 plasmid leptospires. Depicted are the differences between LigAB mutant preparations **(A)**, and LipL32 mutants **(B)** compared with dCas9 alone control samples. The solid line represents an FDR of 0.01 and dotted line represents an FDR of 0.05.

Comparison of the proteome of leptospires containing the empty pMaOri.dCas9 plasmid with those containing pMaOri.dCas9sgLigAB and in which expression of LigAB was not detected identified 46 proteins that were significantly differentially expressed with an FDR of 0.05 (Figure 2A and Supplementary Table 1). Confirming successful silencing, LigB (Q72V39) was not detected in the LigAB group but was found in the LipL32 mutant and dCas9-alone control groups. Whereas LigA (G1UB65) only met the threshold for detection in one of four biological replicates in the dCas9-alone control samples, it was identified in all four LipL32 mutant replicates and was not detected in any LigAB mutant samples. This is consistent with the immunoblots shown in Figure 1B that suggest LigA and LigB are more abundant in the LipL32 mutant compared with the dCas9-alone control. Of the 46 DE proteins between LigAB and dCas9-alone, 27 proteins were less abundant in the LigAB mutants, and 19 proteins were significantly more abundant in the LigAB mutants (see Supplementary Table 1). Proteins with less expression in the LigAB mutant preparations include a surface membrane receptor protein (Q72UE3, LIC10714), a putative lipoprotein (Q72UE4, LIC10713), and bacterial virulence associated Bacterioferritin (Q72SR5, LIC11310) (Oliveira et al., 2020) as well as four uncharacterized proteins [Q72QA5 (LIC12207), Q72NY5 (LIC12693), Q75G04 (LIC20011), Q72W17 (LIC10124)].

Proteins that were more abundant in LigAB mutants included an additional five uncharacterized proteins [Q72MV0 (LIC13089), Q72ST1 (LIC11294), Q72MM7 (LIC13166), Q72S85 (LIC11499), Q72V90 (LIC10411)] as well as the virulence factor surface protein LipL41 (Q72N71) (see Supplementary Table 1). As an additional control, sgRNA off targets (potential regions in addition to the primary target location that could possibly interact with the sgRNA) were determined; for the LigAB mutant it should be noted that the only possible predicted off target to the sgRNA targeting *ligA* and *ligB* was gene LIC11489 (protein Q72S95), which was not affected, further illustrating the high specificity of the CRISPRi approach.

Comparison of dCas9-alone to LipL32 mutant preparations resulted in 243 DE (FDR of 0.05) proteins, illustrating a substantial change in the proteomic profile of the LipL32 mutant leptospires (Figure 2B and Supplementary Table 1). Low trace levels of LipL32 protein (Q72SM7) were still detected in the LipL32 mutant samples compared with the high levels of expression in the dCas9 and LigAB groups [Log2(fold change) = 7.79 difference between LipL32 and dCas9 groups]. There were 159 DE proteins less abundant in the LipL32 mutants compared with the dCas9-alone controls. Decreased expression in the LipL32 mutant leptospires included several lipoproteins [Q72UE4 (LIC10713), Q72T99 (LIC11122), Q72R05 (LIC11947), Q72ND9 (LIC12895)], hemolysin (Q72VH2, LIC10325), Bacterioferritin (Q72SR5, LIC11310), 27 uncharacterized proteins, and the surface membrane protein LipL21. Notably, there were 84 proteins more abundant in the LipL32 mutants compared with the dCas9 control group, including numerous virulence factor and surface membrane proteins, such as LipL45 (Q72RU5), LipL31 (Q72SC8), LigB (Q72V39), and LipL41 (Q72N71). Additionally, more highly expressed in the LipL32 mutants are peptidoglycan-associated cytoplasmic membrane proteins [Q75FE4 (LIC20250), Q72VV5 (LIC10191)], a putative lipoprotein (Q72R63, LIC11885), and 15 uncharacterized proteins (see Supplementary Table 1). Among the upregulated proteins, LIC12339 (Q72PX8, 21-fold increased in LipL32 mutant leptospires compared with dCas9 alone control) and LIC11888 (Q72R60, 19-fold increased in LipL32 mutant leptospires compared with dCas9 alone control) were the most affected (see Supplementary Table 1). Interestingly, both proteins were previously shown to be upregulated when leptospires are cultivated within dialysis membrane chambers implanted into the peritoneal cavities of rats (Caimano et al., 2014). *In silico* analysis indicated that there were only two possible off targets to the sgRNA targeting *lipL32;* LIC10711 [Q72UE6, −1.7 Log2(fold change)] and LIC13432 (Q72LW1, not found in the proteome).

**FIGURE 3:**
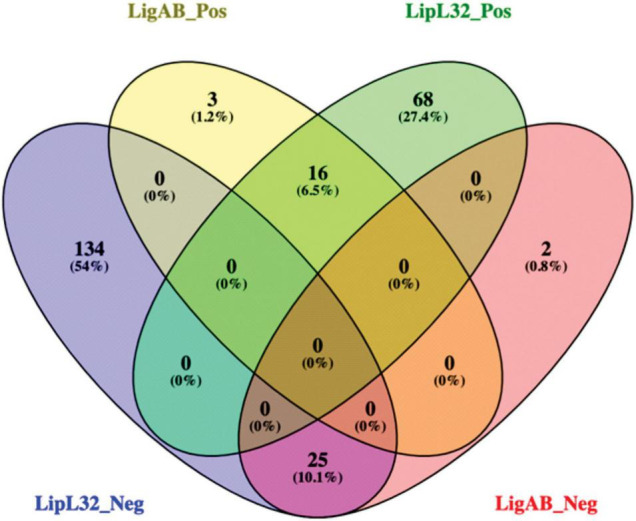
Venn diagram of shared proteins differentially expressed between LigAB and LipL32 mutants compared with empty dCas9 plasmid containing leptospires. Depicted are the number of significant DE proteins (FDR of 0.05) with a positive fold change between LigAB and dCas9-alone (yellow, indicating proteins that were more abundant in the LigAB mutant), negative fold change between LigAB and dCas9-alone (red, indicating proteins that were less abundant in the LigAB mutant). Also shown are the number of significantly DE proteins (FDR of 0.05) with a positive fold change between LipL32 and dCas9-alone (green, indicating proteins that were more abundant in the LipL32 mutant), negative fold change between LipL32 and dCas9-alone (blue, indicating proteins that were less abundant in the LipL32 mutant).

When we compared the DE proteins between LigAB and LipL32 against dCas9-alone, we found conservation of DE proteins both upregulated and downregulated in mutant strains (see Figure 3). There were 25 proteins with lower expression in both the LigAB and LipL32 mutant strains compared with the dCas9-alone control (Figure 3, intersection of negative fold change blue and red groups). These include Bacterioferritin (Q72SR5, LIC11310), a cytoplasmic membrane protein (Q72UE6, LIC10711), outer membrane receptor protein (Q72UE3, LIC10714), a putative lipoprotein (Q72UE4, LIC10713), and four uncharacterized proteins [Q72NY5 (LIC12693), Q72S17 (LIC11570), Q72QX5 (LIC11983), Q72QA5 (LIC12207)]. There were 16 proteins that were increased in both LigAB and LipL32 mutant leptospires compared with dCas9 alone control (Figure 3, intersection of positive fold change yellow and green groups). These include LipL41 (Q72N71), a transcriptional regulator (Q72RX1, LIC11617), and four uncharacterized proteins [Q72ST1 (LIC11294), Q72MM7 (LIC13166), Q72S85 (LIC11499), Q72V90 (LIC10411)].

### LipL32 and Double LigAB Mutants’ Phenotype in Animal Model

**FIGURE 4:**
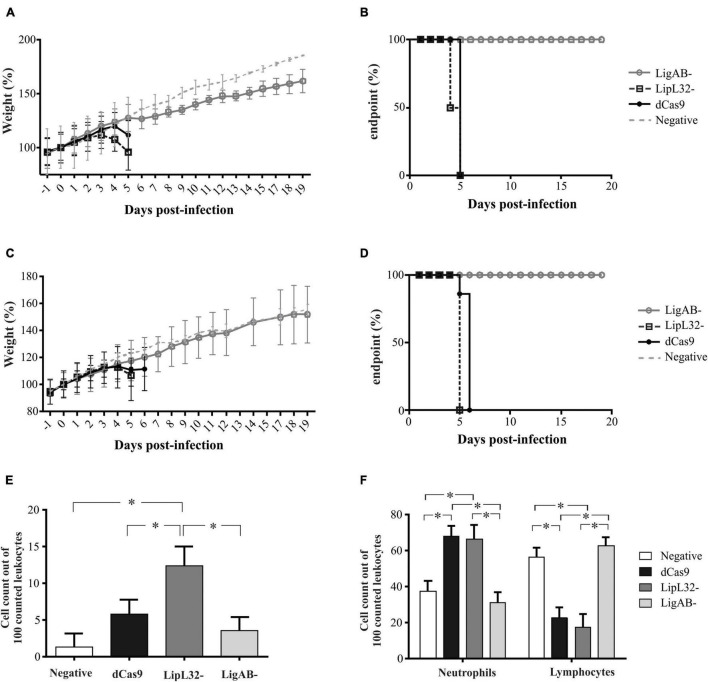
Weight loss, end-point curves, and blood cell counts from infected animals. Hamsters (*n* = 4) were injected intraperitoneally with *L. interrogans* cells (10^8^) containing empty pMaOri.dCas9 plasmid (dCas), mutants for LipL32 or LigAB or media alone (negative control). Animals were monitored daily for weight **(A,C)** and clinical signs of acute leptospirosis, the appearance of which defined endpoints **(B,D)**. Graphics in panels **(A,B)** refer to the first experiment, and in panels **(C,D)** to the second. Panel **(E)** denotes the presence and counts of circulating foamy macrophages, and **(F)** denotes neutrophil and lymphocyte counts among challenge groups. (*) denotes *p*-values ≤ 0.05. Least squares means and standard errors are reported.

Two independent experiments were performed to assess the virulence status of the mutant strains. In the first experiment, each mutant or empty pMaOri.dCas9-containing leptospires were used to IP challenge hamsters (*n* = 4) as a test of mutant strain virulence. When endpoint criteria were met (either severe clinical signs or experimental design; see Table 1), animals were humanely euthanized, liver and kidney tissues were extracted, and macerates were used to inoculate HAN media with or without spectinomycin. All animals infected with the positive control, *L. interrogans* containing pMaOri.dCas9 only, exhibited severe clinical signs of acute leptospirosis, illustrated by prominent weight loss (at day 5), contrasting with no apparent sign of disease or weight loss in the LigAB mutant strain challenged animals (*P* < 0.05) (Figures 4A,B). An endpoint at 4–5 days postchallenge for wild-type *L. interrogans* strain Fiocruz L1-130 is consistent with what has been previously published (Wunder et al., 2021). Asymptomatic hamsters challenged with the LigAB mutant were humanely euthanized 19 days postchallenge consistent with the establishment of a chronic infection. From the LigAB challenged hamster tissues, leptospires could be isolated only from the kidneys, in media with or without spectinomycin (Table 2). Typically, widespread bacterial presence in organs besides the kidney (such as the liver) is consistent with acute disease presentation, whereas colonization limited to the kidney is associated with chronic disease. Collectively these results suggest that LigA and LigB play an essential role in the acute disease phenotype. Notably, animals infected with LipL32 mutants displayed unexpectedly severe clinical symptoms early postchallenge, whereby two animals had to be euthanized at day 4,and two at day 5 (Figures 4A,B and Table 1). All animals from the empty dCas9 and LipL32 mutant challenged groups were culture positive for leptospires in both kidney and liver (macerates were used to inoculated HAN media plus 5-FU with or without spectinomycin) (Table 2). Leptospires were successfully cultured in media without or with spectinomycin, indicating that cells were still harboring the plasmid responsible for gene silencing (Table 2).

**TABLE 2:** Culture results of liver and kidney macerates in HAN media without or with spectinomycin (Spc).

	HAN (positive/negative culture)	HAN + Spc (positive/negative culture)
**Experiment 1**		
pMaOri.dCas9 only (day 5)	Liver (4/0)/Kidney (4/0)	Liver (4/0)/Kidney (4/0)
LipL32 mutant (day 4 and 5)	Liver (4/0)/Kidney (4/0)	Liver (4/0)/Kidney (4/0)
LigAB mutant (day 19)	Liver (0/4)/Kidney (4/0)	Liver (0/4)/Kidney (4/0)
**Experiment 1**		
pMaOri.dCas9 only (day 5 and 6)	Liver (8/0)/Kidney (8/0)	Liver (8/0)/Kidney (8/0)
LipL32 mutant (day 5)	Liver (4/0)/Kidney (4/0)	Liver (4/0)/Kidney (4/0)
LigAB mutant (day 6)	Liver (4/0)/Kidney (4/0)	Liver (4/0)/Kidney (4/0)
LigAB mutant (day 19)	Liver (0/4)/Kidney (4/0)	Liver (0/4)/Kidney (4/0)

For the second animal experiment, mutants recovered from kidneys of hamsters challenged in the first experiment (grown in the presence of the antibiotic and validated for target protein silencing) were used for disease challenge. Using mutant leptospires cultured from animal tissues in the first experiment is a unique strength of our study because it minimizes *in vitro* culture variability and illustrates the repeatability and heightened stringency evaluation of virulence. This approach validates the consistency of the results produced in the first experiment, tests the conserved virulence of mutants from animal host tissue as opposed to *in vitro* where culture attenuation is possible, and allows for additional groups of hamsters to be included for both additional endpoint consideration and timepoint synchronization with other groups. For instance, although we established in the first experiment that LigAB mutant-infected animals develop largely asymptomatic chronic infections, in the second experiment, an additional group was sampled at an acute timepoint concomitantly to the presentation of symptoms and sampling of the pMaOri.dCas9 only positive control for meaningful comparisons. Similarly, because the LipL32 challenged animals had an earlier onset of clinical symptoms compared to the pMaOri.dCas9 only positive control, an additional pMaOri.dCas9-alone Fiocruz L1-130 positive control group was sampled and compared with the LipL32 mutant challenged group (see Table 1). At day 5, all the LipL32 mutant challenged animals and one animal from the dCas9 control reached endpoint criteria and were sampled; at day 6, the remaining animals from the dCas9-alone control were sampled (Figures 4C,D). As seen in the first experiment, no animal from the LigAB mutant infected group displayed acute signs of disease, and groups were sampled on day 6 to compare with pMaOri.dCas9-alone Fiocruz L1-130 positive controls and at day 19 to further characterize the chronic bacterial infection.

All animals that presented with clinical symptoms in the acute time frame (sampled at ≤7 days) were culture positive in both kidney and liver macerates in both media with and without spectinomycin. Both livers and kidneys were also culture positive from the asymptomatic LigAB mutant challenged animals collected at the acute time frame in the second experiment. However, asymptomatic animals challenged with the LigAB mutant strain sampled during a chronic (days 14–22 postinfection) time frame, were only positive for mutant leptospires in their kidneys. In all cases, bacteria could be cultured in media with or without spectinomycin in a similar fashion (Table 2) and, once again, indicating high plasmid stability *in vivo*.

Analysis of whole blood of hamsters recently revealed circulating leukocyte profiles associated with severity of leptospirosis infection (Putz et al., 2021). Notably, these observations included the presentation of foamy macrophages in circulation, which appear to be positively associated with more virulent challenge strains of *Leptospira*. In the present study, blood smears of hamsters challenged with dCas9 alone, LigAB, and LipL32 mutant strains as well as control samples were manually evaluated for leukocyte counts (first 100 white blood cells characterized). Foamy macrophages were identified in all groups (Figure 4E). Consistent with the virulence phenotypes described above, the highest percentage of foamy macrophages appear in the LipL32 mutant challenged animals (12.33 ± 2.64 counts per 100 cells), which was significantly higher than the control group (1.33 ± 1.87 counts per 100 cells, *p*-value < 0.01), the dCas9 alone group (5.82 ± 1.95 counts per 100 cells, *p*-value = 0.05), and the LigAB mutant challenged group (3.42 ± 1.87 counts per 100 cells, *p*-value < 0.01). Consistent with previously published whole blood evaluations, neutrophil counts were significantly elevated (*p*-value < 0.01) in animals challenged with severe disease-causing strains of *Leptospira*, in this experiment, the dCas9 alone and LipL32 mutant challenged groups (67.6 ± 5.87 and 66.3 ± 7.95 counts per 100 cells, respectively) compared with the LigAB mutant challenged and control groups (31.0 ± 5.62 and 37.6 ± 5.62 counts per 100 cells, respectively) (Figure 4F). Also, consistent with the Putz et al. study (Putz et al., 2021), lymphocyte counts were significantly lower (*p*-value < 0.01) in animals challenged with severe disease-causing dCas9 alone and LipL32 mutant challenged groups (22.8 ± 5.34 and 17.5 ± 7.23 counts per 100 cells, respectively) compared with the LigAB mutant challenged and control groups (62.3 ± 5.11 and 56.2 ± 5.11 counts per 100 cells, respectively) (Figure 4F). Monocyte counts showed no significant differences among any groups.

### Quantification of Bacterial Burden in Target Organs

Extracted DNA from kidney cortex and liver tissue were used as a template for qPCR amplification targeting the *lipL*32 gene; concomitantly, a standard curve was included, ranging from 10^7^ to 10^0^ genomic equivalents (GEq) for estimating the bacterial load on target tissues. For comparison, groups from the second experiment concurrently sampled were analyzed. A concentration curve based on *X*-axis values (logGEq) and *Y*-axis (Ct) rendered a slope of −3.43 and *Y*-axis intersection of 38.95 (*R*^2^ = 0.999).

**FIGURE 5:**
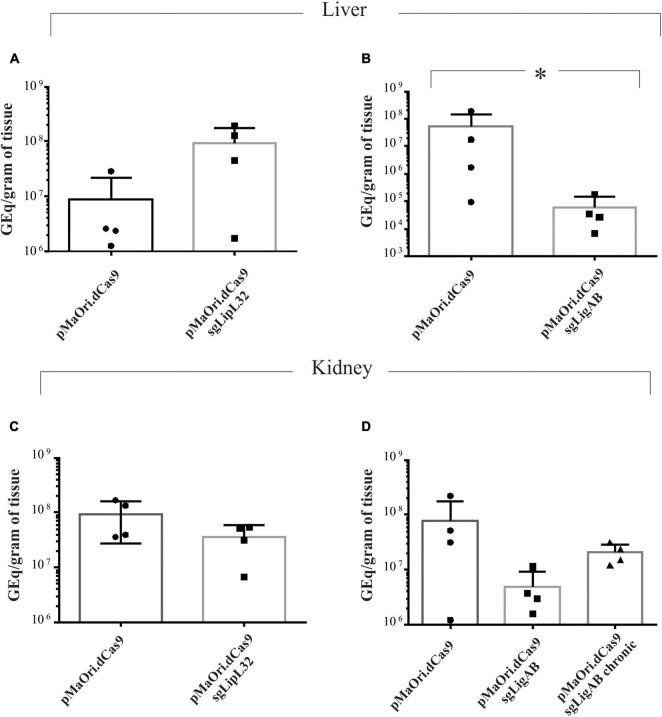
Bacterial burden quantification in target organs from infected animals. Total DNA from liver **(A,B)** and kidney **(C,D)** tissue from time-synchronized euthanized animals were used as template for qPCR amplification targeting the *lipL*32 gene. The bacterial burden from animals infected with each mutant (LipL32, containing the plasmid pMaOri.dCas9sgLipL32, and LigAB, pMaOri.dCas9sgLigAB) were compared with the control in which animals were infected with leptospires containing empty pMaOri.dCas9. The number of genome equivalents (GEq) were estimated based on a concentration curve run in parallel to the experiment. Additionally depicted in panel **(D)** is the quantification in kidneys from chronically LigAB mutant infected animals. (*) denotes *p*-values ≤ 0.05.

Animals challenged with the LipL32 mutant displayed an average of 9.2 × 10^7^ and 3.6 × 10^7^ GEq per gram of liver and kidney, respectively (Figures 5A,C), contrasting to 8.6 × 10^6^ (liver) and 9.4 × 10^7^ (kidney) in the dCas9-alone infected group (*p*-value for liver = 0.1 and kidney = 0.17). Even though LipL32 mutants could more rapidly disseminate in the liver (∼10-fold), statistical significance was not observed. Consistent with the contrast of the disease phenotypes between dCas9-only control and LigAB mutant challenge groups, differences (∼850-fold) in the liver bacterial burden were observed; whereas the dCas9-alone infected group had 5.3 × 10^7^ GEq per gram of liver, the LigAB mutant-infected group had 6.2 × 10^4^ GEq (*p*-value = 0.028) (Figure 5B). In the kidney, an average of 7.7 × 10^7^ compared with only 4.8 × 10^6^ GEq per gram of tissue (*p*-value = 0.17) were found in the dCas9-alone and LigAB-infected groups, respectively (Figure 5D). The LigAB-mutant infected animals in the chronic time frame were negative for the presence of leptospires in the liver and exhibited an average of 2 × 10^7^ GEq per gram of kidney and compatible to the organ macerates’ culture results (Table 2). The initial detection of leptospires in both liver and kidney but subsequent clearing from liver and detection only in kidney is consistent with the dissemination kinetics of leptospires establishing persistent renal colonization (Athanazio et al., 2008).

### Phenotype Stability in Recovered Mutants

**FIGURE 6:**
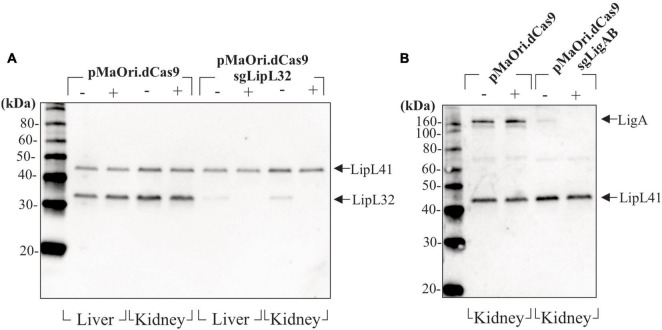
Evaluation of gene-silencing stability after mutants’ recovery. Liver and kidneys from animals sampled after reaching endpoints or at the end of the experiment (chronic infection) were macerated and inoculated in HAN media plus 5-FU in the absence (−) or presence (+) of spectinomycin for selectively allowing mutant growth. Cells recovered from animals sampled at the acute time frame (4–5 days) **(A)** and at the end of the experiment **(B)** were evaluated by immunoblotting.

Leptospires recovered from target organs after infection and grown in HAN media with or without spectinomycin were evaluated for expression of target proteins by immunoblotting. For the acute time frame, cell extracts from *Leptospira* cells containing either pMaOri.dCas9 only or LipL32 silenced protein were compared. As expected, LipL32 levels were similar in the control containing pMaOri.dCas9 only, in the presence (+) or absence (−) of spectinomycin (Figure 6A); as for the LipL32 recovered mutants, from both kidney and liver, the presence of the antibiotic (+) resulted in the retrieval of only mutant cells as denoted by the absence of LipL32 bands (Figure 6A). When the antibiotic was omitted in the macerate’s inoculation on HAN media, only a slight LipL32 signal could be observed, demonstrating plasmid stability *in vivo* (Figure 6A). For the chronic time frame, evaluation of the double LigAB mutants recovered from kidney (no growth was observed in livers) presented a similar result in which only a minor LigA protein signal could be detected when cells were grown without (−) antibiotic (Figure 6B). This similar “leakage” profile of expression in the absence of spectinomycin at distinct time frames led us to conclude that this plasmid loss was likely attributed to the *in vitro* culture rather than from the *in vivo* pressure.

## Discussion

The development and application of the novel CRISPRi methodology has allowed the rapid and straightforward recovery of complete and targeted knockdown mutants in both saprophytic (Fernandes et al., 2019) and pathogenic leptospiral strains (Fernandes et al., 2021). When applied to pathogenic *L. interrogans* serovar Copenhageni strain Fiocruz L1-130, CRISPRi accomplished the silencing of the major LipL32 protein and complete and simultaneous silencing of both LigA and LigB proteins. Previously published pathogenic mutant strains validated the involvement of Lig proteins in serum resistance *in vitro* (Fernandes et al., 2021). Nonetheless, confirmation of mutant strain phenotypes in animal models and whether they would reproduce previous work with transposon knockout *lipL32* (Murray et al., 2009) and transposon-delivered TALE knockdown *ligA/B* (Pappas and Picardeau, 2015) mutants remains to be evaluated. This manuscript sought to further evaluate silenced LigAB and LipL32 mutant strains and assess their proteomic changes and virulence in the context of the hamster model of leptospirosis.

When challenged with LigAB mutants, hamsters developed asymptomatic chronic infections, signifying reduced virulence compared with the dCas9-alone controls. Notably, whereas LigAB mutant leptospires could only be cultured from the kidney during the chronic time frame, they were recovered from both livers and kidneys when LigAB-challenged hamsters were evaluated during an acute (<7 days postinfection) time frame. Consistent with these results, bacterial burden evaluations in LigAB mutant-challenged target organs showed statistically significant and substantially reduced liver colonization by the mutant in comparison with the dCas9-alone positive control (approximately 850-fold decrease), whereas reduced (16-fold) but not significant burden was observed in the kidney. This could be explained by increased susceptibility to complement serum killing by the host and as we previously observed using *in vitro* cultivated mutants in the absence of expression of LigA and LigB (Fernandes et al., 2021) or by other mechanisms not yet elucidated.

Our results demonstrating asymptomatic chronic infection caused by *ligAB* silenced mutants partially agrees with those obtained by Pappas and Picardeau (2015) using serovar Manilae; their incomplete knockdown LigAB mutant also failed to cause acute disease in hamsters, but their mutant could not be recovered from target organs. This discrepancy may be attributed to either the higher inoculum used in the present work or the different serovars used to generate the mutants. Accordingly, although immunization with a LigA/LigB containing chimeric protein could fully protect hamster from lethal challenge with *L. interrogans* serovar Copenhageni strain Fiocruz L1-130, sterilizing immunity was not achieved because leptospires could still be isolated from the kidney of surviving animals (da Cunha et al., 2019).

In contrast, when evaluated by hamster challenge, we observed that the LipL32 mutant-infected animals exhibited severe clinical symptoms and weight loss prior to the onset of the same symptoms in the dCas9-challenged control animals. Consistent with these findings, hamsters challenged with the LipL32 mutants had significant numbers of foamy macrophages, increased neutrophil counts, and decreased lymphocyte counts compared with other experimental groups, which are in agreement with severe leptospiral infections, including the Fiocruz L1-130 strain (Putz et al., 2021). The increased numbers of circulating foamy macrophages in the LipL32 mutant-challenged hamsters suggests a potentially modified interaction of the mutant leptospires with host immune recognition and is of particular interest as the role of this cell type in leptospirosis pathogenesis and the hamster model at large is still being investigated. Bacterial burden analysis showed that the LipL32 mutant more efficiently disseminated in the liver (as shown in Figure 5A) although this variation was not significantly different. When the transposon knockout *lipL32* mutant was previously evaluated by Murray et al. (2009), the author concluded that this major lipoprotein was not required for either acute or chronic infection because the LipL32 mutant challenged animals demonstrated a very similar survival curve to the wild-type serovar Manilae strain. Those results did not include bacterial quantification in target organs with which to compare with the work presented here; however, the endpoint profile and bacterial quantification in target organs from dCas9-alone infected animals strongly agreed with previously published data by Wunder et al. (2021) using the wild-type strain Fiocruz L1-130, indicating that the expression of Cas9 did not impair virulence.

We were interested in exploring the suggested increased virulence in the LipL32 mutant animal challenge work. LPS content as seen in Figure 1 were highly similar between mutant and dCas9 controls; however, whole cell proteome comparisons between these groups showed significant changes (Figure 2). The LipL32 mutant displayed an array of 243 differently expressed proteins (FDR = 0.05). Notably, upregulated in the LipL32 mutant compared with the dCas9-alone control are several major virulence factor and surface membrane proteins, such as LigB, LipL31, LipL45 (Nally et al., 2001), and LipL41. Due to the sheer abundance and highly conserved nature of LipL32 lipoprotein on the membrane of pathogenic leptospires, the loss of such a protein could drastically alter membrane integrity and protein profiles. It is our hypothesis that other lipoproteins and potential virulence factors could be upregulated to fill a niche left by the silencing of LipL32 production and that increased expression of alternative lipoproteins and surface membrane proteins interact with the host immune recognition system and result in a biologically relevant increase in perceived virulence. This event was also demonstrated in *Borrelia burgdorferi*, with which the lack of the major outer membrane protein, OspC, was expressively counterbalanced by the overexpression of other lipoproteins (Xu et al., 2008). The overexpression of LigB (and possibly LigA) in the *lipL32* mutant, as per proteomic data, and both LigA and LigB as per immunoblots, could provide enhanced resistance to complement to account for the increased bacterial burden in liver. LipL32 protein was detected in drastically reduced amounts in the LipL32 mutant; this is similar to previous studies of LipL32 mutants generated by transposon mutagenesis in which low levels of *lipL32* transcripts were detected and most likely a background signal (Murray et al., 2009).

The stability and maintenance of episomal dCas9 and sgRNA plasmids during animal infection were also assessed in this work; at all acute and chronic time points examined, mutant leptospires were recovered from target organs, and functionality of plasmids silencing gene expression confirmed by immunoblotting even when mutants were cultured in the absence of selective antibiotics. This, in turn, validates the applicability of CRISPRi to assess virulence factors *in vivo*. In this work, we establish that the CRISPRi silenced *L. interrogans* LigAB mutant is attenuated and causes a chronic infection in challenged hamsters in the absence of clinical signs but with recoverable leptospires from kidneys of infected animals. Evaluation of the LipL32 mutant resulted in quicker onset of clinical signs and increased bacterial burden in liver compared with the dCas9-alone-challenged animals. We hypothesize that the reorganization and increased expression of surface membrane proteins, including other lipoproteins, contributed to this phenotype and should be further investigated to identify their role in virulence. Collectively, results demonstrate the advantages of using CRISPRi technology to evaluate and characterize virulence and host-pathogen immune interactions of leptospires.

## Data Availability Statement

The original contributions presented in the study are included in the article/Supplementary Material, further inquiries can be directed to the corresponding author/s.

## Ethics Statement

The animal study was reviewed and approved by Animal Care and Use Committee at the National Animal Disease Center, and as approved by USDA Institutional guidelines.

## Author Contributions

LF, EP, JS, JL, AN, and JN: literature revision and manuscript preparation. LF, EP, JL, AN, and JN: experimental design. LF, EP, JS, JL, and JN: experimental manipulation. LF, EP, and JN: figures. All authors reviewed and approved the manuscript, participated in the literature revision, discussion, and preparation of manuscript.

## Conflict of Interest

The authors declare that the research was conducted in the absence of any commercial or financial relationships that could be construed as a potential conflict of interest.

## Publisher’s Note

All claims expressed in this article are solely those of the authors and do not necessarily represent those of their affiliated organizations, or those of the publisher, the editors and the reviewers. Any product that may be evaluated in this article, or claim that may be made by its manufacturer, is not guaranteed or endorsed by the publisher.

## Supplementary Material

Supplementary Figure 1 Representative immunoblots showing the apparent upregulation of LigA and LigB in the LipL32 mutant. **(A–C)** Total of 5 × 10^7^ recombinant cells containing empty pMaOri.dCas9 plasmid alone or with sgRNA for LipL32, and LigAB, were evaluated by immunoblot with anti-LipL32, anti-LipL41 and anti-LigAB polyclonal antibodies. Molecular mass markers (M) (kDa) are indicated.

Supplementary Table 1 Perseus analyzed differential evaluations of proteomic contrasts of interest. Sheets denote all major contrasts of interest (dCas9-alone versus LigAB mutant, and dCas9-alone versus LipL32 mutant). Significantly DE proteins (Perseus distinction with FDR of 0.05) are highlighted in orange, and biological replicates being compared have abundance scores highlighted in yellow. All output data from Perseus DE analysis with all conditions are also provided.
