# Supplementary figures and images for: Evaluation of LipL32 and LigA/LigB Knockdown Mutants in Leptospira interrogans Serovar Copenhageni: Impacts to Proteome and Virulence
Source: Front Microbiol. 2022 Feb 2;12:799012. doi: 10.3389/fmicb.2021.799012 (PMC8847714; doi:10.3389/fmicb.2021.799012)

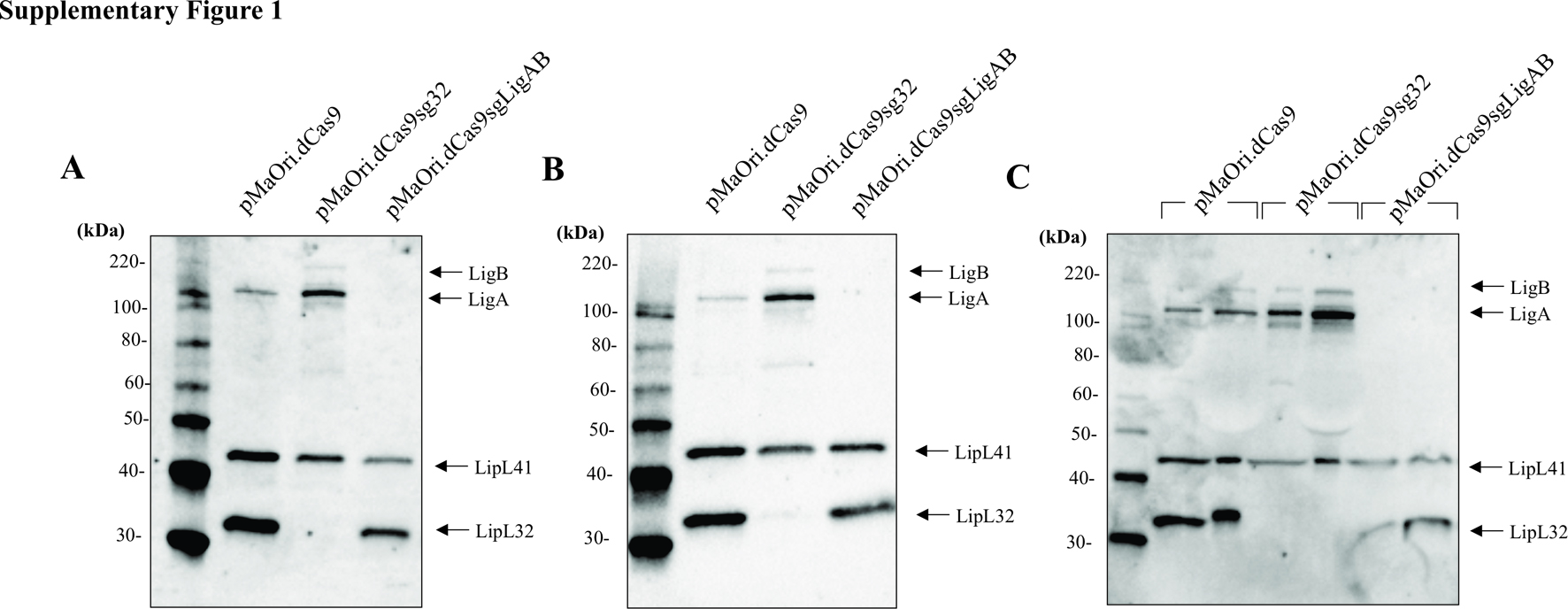

Supplement: Supplementary Figure 1 — Representative immunoblots showing the apparent upregulation of LigA and LigB in the LipL32 mutant. (A–C) Total of 5 × 10^7^ recombinant cells containing empty pMaOri.dCas9 plasmid alone or with sgRNA for LipL32, and LigAB, were evaluated by immunoblot with anti-LipL32, anti-LipL41 and anti-LigAB polyclonal antibodies. Molecular mass markers (M) (kDa) are indicated. [file Image_1.JPEG]
